# Supplementary material for: A multiplex CRISPR interference tool for virulence gene interrogation in Legionella pneumophila
Source: Commun Biol. 2021 Feb 4;4:157. doi: 10.1038/s42003-021-01672-7 (PMC7862264; doi:10.1038/s42003-021-01672-7)
Supplement: Supplementary file 3 — Descriptions of Additional Supplementary Files [file 42003_2021_1672_MOESM3_ESM.pdf]

Descriptions of Additional Supplementary Files

Supplementary Data 1

Description: Cloning plasmids and strains used in this study.

Supplementary Data 2

Description: CRISPRi constructs and strains used in this study.

Supplementary Data 3

Description: Primers used in this study
